# The Grip on Health Intervention to Prevent Health Problems Among Workers With a Lower Socioeconomic Position: A Pilot Implementation Study

**DOI:** 10.1097/JOM.0000000000002826

**Published:** 2023-03-03

**Authors:** Rosanne Schaap, Frederieke Schaafsma, Maaike Huysmans, Emma Vossen, Cécile Boot, Johannes Anema

**Affiliations:** From the Department of Public and Occupational Health, Amsterdam Public Health Research Institute, Vrije Universiteit Amsterdam, Amsterdam UMC, Amsterdam, the Netherlands.

**Keywords:** intervention, occupational health professional, workers, lower socioeconomic position, health problems, implementation, process evaluation

## Abstract

Workers with a lower socioeconomic position (SEP) often face problems on multiple life domains. This study evaluated an intervention to identify and solve problems on multiple life domains, called “Grip on Health.”

**Methods:**

A mixed-methods process evaluation was performed among occupational health professionals (OHPs) and lower SEP workers with problems on multiple life domains.

**Results:**

Thirteen OHPs delivered the intervention to 27 workers. For seven workers, the supervisor was involved, and for two, stakeholders from outside the workplace were involved. Agreements between OHPs with employers often affected implementation. OHPs were essential to help workers identify and solve problems. The intervention increased workers' health awareness and self-control, and led to small and practical solutions.

**Conclusions:**

Grip on Health can support lower SEP workers with solving problems on multiple life domains. However, contextual factors make implementation difficult.

## Abstract 2

This study evaluated an intervention that occupational health professionals can use to support lower SEP workers with solving problems on multiple life domains.

**LEARNING OUTCOMES**

- Learn how occupational health professionals delivered the Grip on Health intervention among lower socioeconomic position (SEP) workers in occupational health practice.
- Discuss the extent to which Grip on Health can reach and fits lower SEP workers with problems on multiple life domains.
- Discuss how the Grip on Health intervention can support lower SEP workers with solving problems on multiple life domains.

Workers with a lower socioeconomic position (SEP) have an increased risk of health problems and thereby premature dropout from the labor market.^1–3^ The Participatory Approach (PA) is a commonly applied intervention to prevent or reduce health risks at the workplace.^4–6^ The PA consists of a stepwise process to identify and solve problems at the workplace through involvement of relevant stakeholders.^7^ Until now, the PA has been implemented among workers in a wide variety of industries and workplace settings, but has not been specifically tailored to the needs of lower SEP workers. Research showed that the PA can positively impact physical and mental health outcomes^4,5^ and is therefore a promising method to prevent health problems among lower SEP workers.

Whereas the PA solely focuses on problems at the workplace, problems outside the workplace also interfere with work functioning and health, and these problems are more prevalent among lower SEP workers.^8,9^ This group of workers also has less problem-solving skills and is often confronted with an accumulation of problems (eg, work-related problems, financial problems, and unhealthy lifestyles), which makes it difficult for them to solve problems on their own.^8,9^ Interventions that support lower SEP workers to solve problems on multiple life domains are therefore expected to be more effective.^10^ For that reason, the focus of the PA was extended to include a broader perspective on health to identify and solve problems on multiple life domains. This intervention is called “Grip on Health.” In addition, the original PA materials were considered too complex for lower SEP workers^11^ and were also adapted to align with the skills of these workers.

Process evaluations are used to understand the feasibility of the intervention and to determine how, for whom, and under what conditions the intervention is applicable in practice.^12^ Occupational health professionals (OHPs) deliver this intervention, and many factors, such as competence and workload of OHPs, can influence implementation.^13^ Process evaluations can provide knowledge on whether the intervention was delivered as intended by OHPs, how they delivered the intervention in practice, and how they perceived its value to support lower SEP workers. In addition, a process evaluation can also provide more knowledge on whether the intervention has reached lower SEP workers as intended and fits this particular group of workers. More knowledge on the implementation of Grip on Health in practice provides relevant insights on how OHPs could support lower SEP workers with solving problems on multiple life domains, in the context of Grip on Health and beyond.

The importance of process evaluations is increasingly being recognized, as implementation data are valuable for understanding how interventions work in real-world settings.^14^ However, a review on process evaluations of workplace health promotion interventions showed that process evaluations mainly focused on what is delivered and on participation levels, rather than how an intervention is delivered, the quality of delivery, and reasons whether or not to participate in the intervention.^13^ To obtain comprehensive, in-depth information on the implementation process, there is a need for systematic approaches in process evaluations, with data on a wide range of components, collected from different perspectives and with different types of methods.^13–15^ Therefore, this study evaluated the implementation process of the Grip on Health intervention in occupational health practice among OHPs and lower SEP workers, using both quantitative and qualitative methods.

## METHODS

### Study Design

The implementation process is evaluated by applying the Medical Research Council (MRC) process evaluation framework.^12^ Following this framework, the process evaluation consists of three parts: implementation (ie, what is delivered and how?), mechanism of impact (how is the intervention perceived and how does it produce change?), and context (ie, how does context affect implementation and outcomes?). The mixed-methods process evaluation was performed between July 2019 and June 2021 by conducting questionnaires, checklists and semistructured (group) interviews among OHPs, semistructured interviews among lower SEP workers who participated in the intervention, and researcher logs. The Medical Ethics Committee of the VU University Medical Center approved the study protocol. OHPs and lower SEP workers signed a written informed consent form before participation.

### The Grip on Health Intervention

The intervention is a conversation method that consists of a stepwise process to identify and solve problems on multiple life domains that affect work functioning, with the involvement of at least one relevant stakeholder. This process is guided by an independent OHP who is in the role of process leader. In this intervention, the PA is used, meaning that the process leader guarantees equivalent and active input of all participants (ie, worker and other stakeholder) in each step of the intervention and generates consensus on the most important problems and solutions. Therefore, the PA is part of the intervention as a method to reach consensus among stakeholders, which is not the same as participatory action research. Participatory (action) research is a methodology to conduct research, in which researchers actively work together with participants to collect data and they may also take actions to improve the problem that is researched.^16^ In this study participatory (action) research is not used as methodology for conducting research but a mixed-methods process evaluation.

In the first step of Grip on Health, the process leader and worker discuss problems on multiple life domains, prioritize problems, and select the most relevant problems. Second, the process leader and worker decide which stakeholder is relevant to involve in the process, either someone inside or someone outside the workplace. In case of problems at the workplace, the supervisor is a relevant stakeholder. In case of problems outside the workplace, a partner, family member, or another health professional may be a relevant stakeholder. Third, the process leader, worker, and stakeholder (if involved) discuss the problems from their own perspective and strive to reach consensus on the most relevant problems. Fourth, the process leader, worker, and relevant stakeholder brainstorm about possible solutions, reach consensus on solutions, and compose an action plan to implement solutions. Fifth, the process leader and worker evaluate the action plan, and if needed, an additional evaluation moment will be planned. For more information on the content of the intervention and the training for OHPs on the methodology of the intervention, see the article on the adaptation of the PA.^17^

### Recruitment

The intervention was delivered by OHPs in occupational health practice. OHPs were recruited through different occupational health services in the Netherlands and associations for OHPs. Through these organizations, they were invited to participate in the Grip on Health training and this study to evaluate the intervention. OHPs could only participate if they had full confidentiality, because OHPs discuss problems on multiple life domains. In the Dutch context, this meant that OHPs needed to be either registered physicians or nurses, or professionals, such as an occupational labor expert, who work under the legal supervision of an occupational physician. OHPs who wanted to participate in the training and this study received a half-day training on how to follow the steps of the intervention. After the training, OHPs signed an informed consent form to participate in this study. If they also wanted to participate in an interview, they signed an informed consent form before the start of the interview. During the training, OHPs received a practical assignment wherein they were asked to apply the intervention in occupational health practice. A couple of months after the training, a follow-up meeting was planned in which OHPs shared their experiences about the practical assignment, reflected on the different steps of the intervention, and on their role as a process leader.

OHPs delivered the intervention to lower SEP workers who were employed in organizations in which OHPs were working as a health professional. OHPs were asked to deliver the intervention preventively, meaning that workers could already have problems on multiple life domains, but were not called in sick or were on short-term sick leave (ie, <6 weeks). Furthermore, lower SEP workers were all Dutch citizens, legally employed in a Dutch organization, and with at least a permanent or fixed contract of more than 12 hours per week. OHPs delivered the intervention among lower SEP workers in case they noticed that workers had problems on multiple life domains that affected their work functioning or had a high degree of sickness absence. This means that lower SEP worker were recruited by OHPs as part of their normal way of working. Therefore, consent of the worker was not needed. OHPs only asked workers for consent to be approached by a researcher to schedule an interview. If a worker was willing to participate in an interview, then the worker signed an informed consent form before the start of an interview.

### Data Collection

The process evaluation among OHPs was conducted with mixed (quantitative and qualitative) methods during and after implementation of the intervention by means of (1) questionnaires at the end of the training, which were completed by 35 OHPs; (2) checklists directly and 3 months after completion of the intervention, which were completed 27 times for workers who received the Grip on Health intervention; (3) semistructured group interviews during implementation with 13 OHPs who delivered and not (yet) delivered the intervention; (4) semistructured interviews after implementation, with 10 OHPs who delivered the intervention and 3 OHPs who did not implement the intervention; and (5) researcher logs during implementation of the intervention. The process evaluation among participants of the intervention was performed by conducting semistructured interviews with seven lower SEP workers who participated in all steps of the intervention. The checklists for OHPs and interview guides for OHPs and lower SEP workers can be found in Supplemental Digital Content, Additional Files 1, http://links.lww.com/JOM/B311, and 2, http://links.lww.com/JOM/B312. The framework of the MRC was further executed by the use of the model of Steckler et al^18^ and Carroll et al..^19^ Implementation was measured by reach, dose delivered and fidelity at OHP level, and quality of delivery at both OHP and participant levels. Mechanisms of impact were measured by responsiveness and program differentiation, at OHP and participant levels. Context was measured by investigating factors that affect implementation on the level of participants, OHPs, and the intervention itself (ie, design and content of the intervention) and were part of the process evaluation components described previously. This means that results of context are not displayed separately but integrated in the process evaluation components. Contextual factors on organizational and sociopolitical levels were described elsewhere.^9^ For further operationalization of the MRC framework, see Table 1.

**TABLE 1:** Operationalization of the Medical Research Council Framework

Key Component in Relation to Context	Component	Operationalization	Method	Level
Implementation	Reach	Amount and characteristics of OHPs that delivered the intervention and of participants that received the intervention, OHPs suitable to provide the intervention and reasons of OHPs whether or not to provide the intervention	Checklists, logs, and interviews	OHP
	Dose delivered	Amount of intended intervention steps delivered, whether this is feasible and which factors play a role in the delivery of the intervention	Checklists, interviews	OHP
	Fidelity	The extent to which OHPs discuss problems on multiple life domains, guide workers with actively prioritizing and identifying problems and solutions, and involve relevant stakeholders	Checklists, interviews	OHP
	Quality of delivery	The extent to which workers were satisfied with the process leader and the extent to which OHPs are in the role of process leader, meaning that the worker is able to identify and prioritize problems and solutions, create a confidential/safe environment, acknowledge all perspectives, remain impartial, and generate consensus in case a stakeholder is involved	Interviews	OHP, participant
Mechanisms of impact	Responsiveness	Perceived satisfaction about the intervention, materials of the intervention and perceived effectiveness	Checklists, interviews	OHP, participant
	Program differentiation	Unique aspects of the intervention that are perceived essential and contribute to positive effects	Interviews	OHP, participant

### Data Analysis

Quantitative data were analyzed using descriptive statistics. Qualitative data were audiotaped and transcribed verbatim. The analysis started with re-reading the transcripts, listening to audiotapes, and making summaries of each transcript to become familiar with the data. Subsequently, textual segments were inductively open coded by the first coder (R.S.) to produce an initial list of codes indicating the content of the textual segments. Another coder (E.V.) read two transcripts and also performed open coding. The codes of these two transcripts were compared and discussed between the first and second coders (R.S., E.V.) to reach consensus on the codes. Next, codes were deductively categorized according to the different process evaluation components, as were described in Table 1. An overview of codes can be found in Supplemental Digital Content, Additional File 3, http://links.lww.com/JOM/B313.

## RESULTS

### Evaluation of the Training of OHPs

Between July 2019 and October 2020, six sessions of the training were provided to 36 OHPs. See Table 2 for the main characteristics of these OHPs. Two of these sessions were provided online because of the COVID-19 pandemic. The training was rated, on average, 8.2 on a scale from 1 to 10. Role playing and the possibility to interact with each other were rated most positive. Suggested improvements for the training related to more practice time for role playing and to the relevance of provided information, as for some OHPs, not all information was new.

**TABLE 2:** Characteristics of Occupational Health Professionals Who Participated in the Training

Characteristics		*n*
Employed by	Employed by an occupational health service	32
	Self-employed	4
Profession	Absenteeism consultant/employability coach	10
	Occupational nurse/employability coach	7
	Occupational physician	6
	Work ability specialist	6
	Occupational labor expert	4
	Occupational social worker	2
	Return to work coordinator	1

### Implementation of the Grip on Health Intervention

In the following section of the Results, we will describe implementation (ie, what is delivered and how?) by reach, dose delivered, fidelity, and quality of delivery, taking contextual factors into account that may affect or affected implementation of Grip on Health.

#### Reach

Thirteen OHPs delivered the intervention in practice. These professionals were absenteeism consultants or employability coaches (*n* = 3), occupational nurses or employability coaches (*n* = 3), work ability specialists (*n* = 2), occupational social workers (*n* = 2), occupational physicians (*n* = 2), and one occupational labor expert (*n* = 1). Twenty-three OHPs did not deliver the intervention in practice. These professionals were absenteeism consultants or employability coaches (*n* = 7), occupational nurses or employability coaches (*n* = 4), work ability specialists (*n* = 4), occupational physicians (*n* = 4), occupational labor expert (*n* = 3), and one return to work coordinator (*n* = 1). The main reasons for OHPs to not deliver the intervention are described in Box 1.

Box 1 Main reasons for OHPs to not deliver the intervention

**Table:** 

Reason	*n* (reason mentioned by OHPs)
Mainly in contact with higher SEP workers or with workers on long-term sick leave in daily practice	10
Lack of time (for multiple consultations)	9
Lower SEP workers with problems on multiple life domains are difficult to reach (preventively) in daily practice	7
No permission from contracted employer, due to other priorities or other comparable interventions in practice	5
Solely conducts consultations by telephone, partially due to COVID-19 pandemic	4
No time to (preventively) reach workers or no request for (preventive) consultations, due to the COVID-19 pandemic	3

In total, 27 workers received the Grip on Health intervention. The main characteristics of these workers are described in Table 3. Although the focus of our study was on lower SEP workers, OHPs stated in the interviews that this intervention is also relevant for high SEP workers, as they may also face problems on multiple life domains and may find it difficult to solve these problems.

**TABLE 3:** Characteristics of Participants in the Intervention

Characteristics		*n*
Type of occupations	Administrative related (eg, secretary worker)	7
	Manufacturing related (eg, production worker)	10
	Service related (eg, service desk, kitchen worker)	4
	Health related (eg, home care worker)	2
	Unknown	4
	Blue-collar occupation	14
	Non–blue-collar occupation	13
Type of contract	Number of hours according to contract	Mean, 35.3 (24–40) h
Sex	Man	15
	Woman	12
Age	<35 y	7
	35–55 y	13
	>55 y	7
Chronic disease	Yes	14
	No	13

OHPs reported that the intervention could be delivered by any type of OHP. Some reported that particularly occupational social workers are most suitable to deliver this intervention, as they already discuss problems on multiple life domains in their daily practice. However, others reported that this intervention could also be helpful for OHPs who usually do not discuss problems on multiple life domains. Several OHPs, including OPs themselves, mentioned that OPs are less suitable to deliver this intervention because of a lack of time. Thus, other professionals with more time, such as occupational nurses, seem to be more suitable to deliver the intervention, as one OHP mentioned in an interview:

OHP1: We as occupational nurses have an hour or one hour and a half, while you only have a maximum of half an hour at the doctor's office, and occupational nurses are therefore very suitable, from my perspective, to make the connection between the medical and private perspective.

Some OHPs mentioned that professionals outside occupational health care, such as social workers or general practice nurses, could also deliver the intervention, as they are better able to reach lower SEP workers with problems on multiple life domains. However, in case there are problems at the workplace, it is important that these professionals refer workers to OHPs or collaborate with them.

##### Dose Delivered

OHPs needed, on average, three to four consultations to deliver the intervention. Among 16 workers, all intended intervention steps were delivered. Step 6: solution analysis, step 7: action plan, and step 8: evaluation were delivered the least because workers were not willing to continue; the intervention led to undesirable results for the worker; the worker and employer were unable to come to an agreement or had a conflict; or the OHP was not involved in these steps. In the interviews, half of the OHPs reported they had insufficient time to deliver the intervention as intended. Discussing problems on multiple life domains can take a lot of time, as was stated by an OHP:

OHP4: For that part you actually need an hour according to this method, and I only had half an hour. Then you just find out that to discuss problems on multiple life domains, you can't do that in half an hour. So, I had to do that in two parts.

Moreover, several OHPs reported that they needed to ask permission in advance from the involved employer to deliver this type of intervention, meaning sufficient consultation time or being able to involve a stakeholder at the workplace. In contrast, the other half of the OHPs reported that they had sufficient time, as they do not need to ask or already have permission from contracted employers or from their own occupational health service to deliver interventions, such as Grip on Health. Some OHPs also reported that they gained trust from contracted employers to organize their own time for a consultation or that the intervention was comparable to their normal way of working, also meaning that they had sufficient time. OHPs also reported that involved employers who recognize the potential value of prevention and sustainable employability for their employees provide OHPs more consultation time, and they are more willing to involve a stakeholder at the workplace in the intervention.

#### Fidelity

The checklists showed that for the majority of the workers, problems and solutions were identified for both inside and outside the workplace (Table 4). Several OHPs stated in the interviews that the discussion of problems on multiple life domains was self-evident and part of their normal way of working. Most solutions that were suggested in the intervention were implemented, and implementation was mostly performed by workers themselves.

**TABLE 4:** Identification of Problems and Implementation of Solutions

Problems and Solutions		*n*
Type of problems	In the workplace	26
	Outside the workplace	23
	Both inside and outside the workplace	22
Discussed problems in the workplace	Problems related to job content	14
	Problems related to working environment	16
	Problems related to working conditions	5
	Physical health problems in the workplace	10
	Mental health problems in the workplace	13
	Lifestyle-related problems in the workplace	4
	Socially related problems in the workplace	11
Discussed problems outside the workplace Type of solutions	Physical health problems outside the workplace	7
	Mental health problems outside the workplace	16
	Lifestyle-related problems outside the workplace	9
	Socially related problems outside the workplace	15
	For problems in the workplace	24
	For problems outside the workplace	20
	For both problems inside and outside the workplace	19
Number of solutions	Implemented	48
that were implemented	Not implemented/unknown	8
Implemented by	Worker	21
	Supervisor	1
	Worker and supervisor	11
	Worker and professional from outside the workplace	3
	Worker and partner	1
	Unknown	11

The checklists showed that among only seven workers, supervisors were involved as a stakeholder in the intervention. However, in the interviews, several OHPs stated that involvement of supervisors in general takes place very often but coincidentally did not happen during the intervention. Consultations of OHPs with a worker and supervisor are often part of their normal way of working. OHPs stated that supervisors can provide different insights into the problems of the worker in the workplace, and if workers and supervisors jointly identify and reach consensus on solutions, it increases the chance that solutions are actually implemented (faster) at the workplace:

OHP4: In a conversation with the supervisor they search for solutions together, it isn't something that is enforced from the outside. It becomes something of their own and eventually a sort of psychological contract where they feel bound to each other to implement the actions. So, the chance that it will be carried out is much higher.

There were also OHPs that did not involve supervisors in consultations. One OHP described that involving a supervisor implies that workers' problems affecting their work functioning come to the surface, which could lead to negative outcomes such as not extending temporary contracts. Other reasons mentioned by OHPs not to involve supervisors were as follows: (1) supervisors are never involved in consultations, but only managers of supervisors or human resource case-managers; (2) supervisors themselves conduct preventive consultations and OHPs only with workers on sick leave; (3) supervisors are unavailable because of a lack of time; (4) supervisors do not see the added value; (5) workers discuss problems with the supervisor themselves or OHPs notify supervisors on what was discussed; (6) consultations were online or OHPs were not physically present at organizations; or (7) there was a conflict between the worker and supervisor. The checklists showed that in only two cases, a stakeholder from outside the workplace was involved. This was also highlighted during the interviews, as OHPs stated that stakeholders from outside the workplace are sometimes involved and not as often as supervisors. These stakeholders are often spouses who may provide extra information on the situation of workers at home or could positively influence implementation of solutions, as was described by an OHP:

OHP2: I also notice that it has been discussed at home with their partner, and that in certain situations the partner tells me that the two of them will work on it together, but then I think something will actually happen.

However, OHPs stated that involvement of spouses could also hinder the implementation of solutions. For instance, they could control the process and outcome of the conversation or the conversation is used to discuss relationship problems. Furthermore, OHPs stated that a professional from outside the workplace such as general practitioners or social workers is never involved in their consultations, and doing so is reported as complex. Involvement of other professionals solely implies requests for information about the workers' health or referrals. Some OHPs stated that collaboration could be helpful to avoid conflicting advice for the worker.

#### Quality of Delivery

In the interviews, some OHPs stated that the role of the process leader is not difficult, as it is part of their normal way of working, and lower SEP workers are able to identify problems, solutions, or both, but occasionally need support. In contrast, there were also OHPs who perceived the role of process leader as difficult, because they reported that lower SEP workers are less able to identify problems, solutions, or both on their own and need much support. OHPs reported that lower SEP workers have many different problems, leading to a stressful situation, which makes it difficult to disentangle their problems. This is in line with what was described by the participants, as the majority stated that they were satisfied with the OHP because they listened well to their problems and thought along to identify problems, solutions, or both. OHPs also mentioned that self-control is more difficult for lower SEP workers. They are used to professionals telling them what to do, and are less used to take on an active role, to reflect on their problems and on what they themselves can do to solve their problems:

OHP4: People of this target group are not used to talk about their problems, to take self-control, and to discuss solutions with the supervisor, because these are topics that you don't show off with, make you vulnerable, make you ashamed, or which is difficult to talk about.

As a result, OHPs stated that it is difficult to convince lower SEP workers to take on an active role and to make them aware of their own role in solving problems. An important condition for self-control stated by OHPs is that the worker sees his or her own role in solving problems. In contrast to lower SEP workers finding it difficult to take on an active role, OHPs may not always give workers the opportunity to take on an active role. OHPs stated that they are used to take on the role of the expert. If workers themselves come up with solutions, OHPs sometimes have to refrain themselves to give their opinion on the feasibility of solutions. Some OHPs stated that they first gave workers the opportunity to experience whether a solution works. If not, OHPs can always advise workers on other solutions. In addition, workers do not always have an overview of possible solutions. In these cases, OHPs stated that they provided several possible solutions workers could choose from.

Participants reported that they were satisfied about their consultations with OHPs. Participants felt that they were in a safe and confidential environment, wherein they could talk openly about their problems inside and outside the workplace. OHPs communicated in a good and pleasant way, participants felt understood and supported by OHPs, as was described by one participant:

P2: He actually listened very carefully to what was going on and he thought along very well with solutions. So yeah that was very nice.

In case supervisors were involved in the intervention, OHPs stated that they obtained a safe and confidential environment and equality between the worker and supervisor, and reached consensus on problems and solutions. Participants perceived the involvement of supervisors as positive, because they were able to inform the supervisor about their problems and problems were solved faster. However, OHPs mentioned that this is dependent on the relation between workers and supervisors. In case of a good relation, workers are more willing to share their problems. If this is not the case and there is a lack of trust between a worker and supervisor, to obtain a safe and confidential environment is difficult. OHPs also stated that the hierarchical relation between the worker and supervisor is not always easy to change, and they need to be open to a different role.

### Mechanisms of Impact

In the following section, we will describe mechanisms of impact (ie, how is the intervention perceived and how does it produce change?) by responsiveness and differentiation, taking contextual factors into account that may affect or have affected mechanisms of impact of Grip on Health.

#### Responsiveness

**FIGURE 1:**
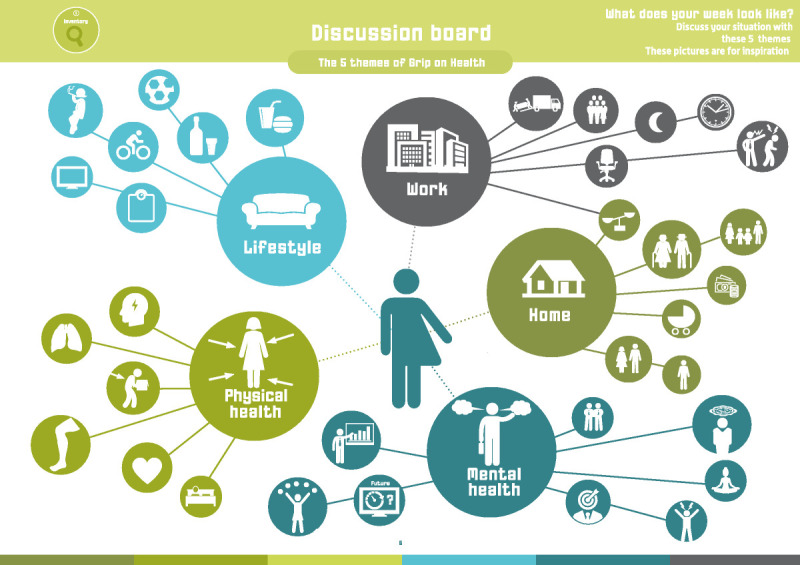
Visual map to discuss problems.

Both OHPs and participants mentioned in the interviews that the intervention is structured, clear, and according to OHPs relevant for lower SEP workers. Several OHPs stated that the intervention is comparable to their normal way of working, but a helpful tool to conduct consultations and to reassure that all steps are performed. Both OHPs and participants were positive about the visual materials of the intervention, as it was a useful tool to discuss and identify problems on different life domains. However, OHPs stated that they mainly used the visual map to discuss problems (Fig. 1). The other materials were perceived too difficult for lower SEP workers, as these contained writing assignments and relied too much on problem-solving skills.

The intervention was perceived useful by most participants and OHPs, as the intervention could have positive effects on health, sick leave, or functioning of workers, which was also presented in the results of the checklists (Table 5). In contrast, some other OHPs mentioned that it is uncertain whether the intervention leads to positive effects. However, both participants and OHPs mentioned that the intervention increased workers' awareness of their health and own role in solving problems, which motivates them to reflect on what they themselves can do to improve their health, as was described by an OHP:

**TABLE 5:** Reponsiveness

		Mean
Satisfaction*	Satisfaction process	4.07
	Satisfaction effectiveness	4.00
	Satisfaction of the worker	3.88
Perceived effectiveness^†^	Health	3.64
	Work functioning	3.43
	Working conditions	2.86
	Living conditions	3.56
	Self-control	3.52
	Support workers on solving problems	3.18
Solutions^‡^	Solutions implemented	2.45

		*n*
Type of problems solved in the workplace	Job environment–related problem	4
	Job content–related problem	4
	Physically related problem at work	5
	Mentally related problem at work	6
	Socially related problem at work	3
Type of problems solved outside the workplace	Physically related problem outside the workplace Mentally related problem outside workplace	2 7
	Lifestyle related problem outside the workplace	3
	Socially related problem outside the workplace	9
Sick leave	Prevented sick leave	4
	Decreased duration of sick leave	13
	Not decreased duration or prevention of sick leave	5

*Scale: 1, very unsatisfied; 5, very satisfied.

^†^Scale: 1, not at all; 5, to a very large extent.

^‡^Scale: 1, all solutions implemented; 5, no solutions implemented

OHP7: Well, I think that this method helps people to become aware of what they could change. Initially to become aware of it, to become self-conscious of what I actually face? Which problems emerge? And then to make them aware of what they could change to actually achieve an improvement of the situation.

OHPs and participants also described that the intervention led to small and practical solutions, which will, according to OHPs, not immediately lead to large effects but a higher chance of solutions being implemented and workers experiencing success. This was also shown in the results of the checklists, as solutions were to a reasonable extent implemented (Table 5).

Although OHPs were reassured that this intervention could lead to positive effects, they also reported that this depends on the worker himself/herself and on external factors inside or outside the workplace. Initially, the worker must be open to change and see their own role in this process. If the worker does not see the problem or is not willing to take on an active role, it is likely that the intervention is less effective. Moreover, OHPs mentioned that some involved employers are not always willing to cooperate in the implementation of solutions or to pay for a solution resulting from the intervention. Finally, social pressure of colleagues or from the social environment of workers at home may also hamper the implementation of solutions.

#### Program Differentiation

OHPs and participants of the intervention reported several essential intervention components that may contribute to positive effects. First, OHPs and participants expressed that the intervention provided an overview of all life domains, which provides workers more insight into (underlying) problems. As a result, workers became aware of problems they did not see themselves, or of problems that influenced their work functioning, as was described by a participant:

P4: I thought it was primarily about the panic attack, but she asked me questions and she talked about certain things more deeply and then a completely different issue came up, which played a role on the background for a long time and the panic attack was an expression of that, and because she asked good questions, this came up all of a sudden.

Second, OHPs and participants described that the structured method and visual materials helped workers to actively discuss problems and to get an overview of their problems. Finally, workers are in the lead to identify problems and solutions, which improves their feelings of self-control and a higher chance that solutions are being implemented.

## DISCUSSION

The aim of this study was to systematically evaluate the implementation process of the Grip on Health intervention in occupational health practice among OHPs and lower SEP workers. Grip on Health can be used to identify and solve problems on multiple life domains among lower SEP workers. Both OHPs and lower SEP workers were satisfied about the intervention and in particular with visual materials of the intervention, as this helped workers to actively discuss and identify their problems. However, many OHPs also experienced difficulties to deliver Grip on Health in occupational health practice.

Many OHPs, including those who delivered the intervention, reported difficulties to preventively reach lower SEP workers, which was also described in other interventions.^20^ OHPs who succeeded to reach lower SEP workers in this study indicated that the intervention was often initiated by the employer or was part of a preventive occupational health examination or absenteeism consultation. Thus, it seems that lower SEP workers do not tend to visit an OHP on their own initiative. OHPs in this study stated that familiarity of the preventive role of OHPs is low, which is in line with findings of another implementation study.^21^ OHPs in this study described that any type of OHP could deliver this intervention, as most OHPs already discuss problems on multiple life domains and it is part of their normal way of working. Moreover, the group of OHPs who did and those who did not deliver the intervention both consisted of a variety of professions. However, discussing and solving problems on multiple life domains can take a lot of time, which was not always available in practice, as was mentioned as one of the reasons to not deliver the intervention. The lack of time experienced by some OHPs often relates to agreements between OHPs and involved employers about the duration of their consultation time. Furthermore, no permission from contracted employers to deliver the intervention was also one of the main reasons to not deliver the intervention. A review on health promotion programs in the workplace showed that management support was the most frequently reported facilitator for delivering interventions.^13^ In the Netherlands, employers pay for, and therefore largely determine, the content and extent of occupational health services provided. In addition, a context analysis for implementation of preventive interventions that consider multiple life domains showed that not all employers feel primarily responsible for solving problems on multiple life domains and still invest too little in prevention.^9^

Findings of this study also showed that implementation of the intervention was (very) limited. One contextual factor that has probably played a role is the COVID-19 pandemic and the increased use of online consultations instead of face-to-face consultations. Moreover, OHPs who succeeded to deliver the intervention could not always deliver all intended steps because of the online consultation sessions. In line with this, another study evaluating Grip on Health among OHPs and general practitioners showed that it was not feasible to use the materials in an online meeting.^22^ Furthermore, during the COVID-19 pandemic, stakeholders at the workplace may have had other priorities than to support preventive interventions focused on multiple life domains. Involving stakeholders at the workplace with lower SEP workers is in general considered difficult, because they do not always have time or see the added value of preventive interventions, as was described in this study and in the literature.^23,24^ It is, however, difficult to conclude that the implementation of Grip on Health either was successful or has failed. In implementation science, there has been a debate about the balance between fidelity (ie, intervention is delivered as intended) and the need for adaptation (ie, changes in the intervention to fit the context).^25^ The results of this study showed that, for some parts, adaptation to the intended intervention was valid. OHPs often had good reasons for not delivering all intervention steps: for example, workers who were not willing to continue or who were not able to come to an agreement with their supervisor, although readiness to participate and having an open mind are preconditions to participate in this participatory intervention.^7^

Involving professionals from outside the workplace in solving problems was considered too complex by the OHPs in this study. This is probably related to the strict separation in the Netherlands between occupational and curative health care, which makes collaboration difficult between professionals from inside and outside the workplace.^9^ Moreover, the literature suggests that skills of OHPs to involve stakeholders play an important role, and training OHPs in involving stakeholders would be useful.^26^ For instance, a study on involving significant others, such as the partner, showed that OHPs have an important role in informing workers about the possibility to involve significant others.^27^ This kind of skills was not addressed in the Grip on Health training and could therefore also have played a role in the limited involvement of stakeholders outside the workplace.

In this study, the role of the process leader was perceived as challenging by most OHPs. They are used to take on the role of the expert and to provide advice to workers on how to solve their problems. Moreover, OHPs described that lower SEP workers find it difficult to take self-control, as they are less able to identify problems, solutions, or both on their own. However, both OHPs and workers in this study stated that the intervention was perceived as effective, mainly because of increasing workers' awareness of health problems. Increasing awareness is the first stage in the transtheoretical model of change.^28^ This is called the precontemplation phase, wherein people do not intend to act and they are often unaware of their problems. This study showed that the intervention provided more insight into problems by discussing different life domains. This is very helpful, as the literature shows that lower SEP workers may have a lower awareness and risk perception of their health problems.^8,17^ Moreover, people with problems on multiple life domains are often in a state of chronic stress, wherein they are unable to oversee their problems.^29^ As a result, people may find it more difficult to be aware of problems and could use passive or avoidant coping styles toward their problems. This may underline the finding in this study that the intervention was also perceived relevant for higher SEP workers. For instance, another study that evaluated Grip on Health also found that this intervention could be applied to a wider group of people.^22^ People with problems on multiple life domains, and especially people with psychological health complaints, have less structure and overview, which temporarily affects their problem-solving skills. These findings may indicate that it is not about the classification of groups into a lower or higher SEP, but about the circumstances in which people live.^30^

In the study about the development of the intervention,^17^ the Self Determination Theory was selected as a theory to enable lower SEP workers to actively identify and solve problems, and may further clarify why both OHPs and workers perceived the intervention to be effective. This theory argues that by increasing autonomy, competence, and relatedness, health-related behaviors are more likely to be initiated and maintained, and thereby, motivation of workers to actively solve their problems is increased.^31^ The need for autonomy, competence, and relatedness could all be identified in the findings of this study regarding mechanisms of impact. Autonomy may have been fulfilled, as both OHPs and participants described that this intervention made workers more aware of the problems they could intervene on, and that workers were in the lead to identify the most relevant problems and solutions, which could improve their feelings of self-control. Competence may have been fulfilled, as OHPs described that the intervention led to small and practical solutions, which in turn increases one's belief in the ability to succeed.^32^ Finally, relatedness of workers may have been fulfilled by a supportive environment of OHPs or other stakeholders to solve their problems.

### Strengths and Limitations

A strength of this study is the use of a comprehensive framework to evaluate the implementation process, which resulted in detailed information about implementation of the Grip on Health intervention in practice. Although Grip on Health was developed using an intervention mapping protocol,^17^ this study provided additional information on the applicability and feasibility of this intervention in practice. Moreover, data were collected from both the perspective of OHPs and lower SEP workers, and a combination of quantitative and qualitative data collection was performed, increasing the credibility of findings.^33^ The data from interviews helped to interpret the results of the checklists or to ensure that findings of the checklists are grounded in the experiences of OHPs and lower SEP workers about the intervention. A limitation of this study is that a large proportion of the data on lower SEP workers was collected through OHPs. OHPs may hold different views on the intervention than lower SEP workers themselves,^34^ affecting the credibility and transferability of findings.^33^ However, the contextual factors affecting the implementation of Grip on Health in this study were also found in other studies,^9,22^ suggesting good transferability and confirmability of findings. Another limitation is that selection bias may have occurred. Lower SEP workers who were interviewed in this study all participated in the Grip on Health intervention and were mainly positive about the intervention. We failed to recruit lower SEP workers who did not participate in the intervention to obtain a more complete view of the experiences of lower SEP workers. This means that it is debatable whether data saturation took place for the qualitative data among lower SEP workers, affecting the dependability of findings.^33^ This was not the case among OHPs; both those who delivered and those who did not deliver the intervention were interviewed, and data were collected until no new themes emerged in the interviews. Unfortunately, no information on OHP characteristics (e.g., sector and size of organization) was collected. This could have given more insight into facilitators and barriers for implementing Grip on Health.

### Implications for Research and Practice

For OHPs to successfully deliver the intervention, it is important that they are able to preventively reach lower SEP workers for the Grip on Health intervention. From this study, we learned that OHPs should make use of additional methods, for example, preventive health examinations, to reach workers preventively. Some OHPs in this study stated that health professionals in curative health care could also deliver this intervention, because they are better able to reach lower SEP workers with problems on multiple life domains. General practitioners are often the first health professional for workers to discuss their health complaints, and workers make little use of the opportunity to visit an OHP preventively.^9^ Therefore, further research should explore how professionals from outside occupational health care can play a role in preventively reaching lower SEP workers or on how they can deliver this intervention. The MRC framework describes that context is one of the main aspects that affect implementation of interventions.^12^ In this study, factors on organizational and socio-political level made it difficult for OHPs to deliver the intervention in occupational health practice. A hindering factor for implementation is that employers eventually determine whether preventive interventions, such as Grip on Health, are delivered to workers. Hence, for OHPs to be able to deliver the intervention and to have sufficient time, cooperation or permission from the involved employer is essential. Another hindering factor is the strict separation in the Netherlands between occupational and curative health care, which caused difficulties for OHPs to involve professionals from outside the workplace in the intervention. To effectively solve problems on other domains than work, collaboration with professionals from outside the workplace may be needed. Hence, further research is needed on how this collaboration could be improved. This study also showed that lower SEP workers find it more difficult to take self-control. The Dutch government and society encourage workers to take self-control for health and sustainable employability.^35^ Lower SEP workers need adequate support from OHPs. However, OHPs in this study experienced difficulties with their role as a process leader, as they are used to take on the role of the expert and workers find it difficult to identify problems, solutions, or both on their own. Hence, education of OHPs needs to focus more on how to enhance self-control among (lower SEP) workers.

## CONCLUSIONS

This study showed that Grip on Health can be a successful method to support lower SEP workers with solving problems on multiple life domains. However, many OHPs found it difficult to deliver the intervention in daily practice, mainly because of contextual factors. Successful implementation of this intervention in occupational health practice could be improved by more research on how to effectively tackle contextual factors.

## Supplementary Material
